# The Impact of Microwave Annealing on MoS_2_ Devices Assisted by Neural Network-Based Big Data Analysis

**DOI:** 10.3390/ma17133373

**Published:** 2024-07-08

**Authors:** Xing Su, Siwei Cui, Yifei Zhang, Hui Yang, Dongping Wu

**Affiliations:** 1 State Key Laboratory of ASIC and System, Fudan University, Shanghai 200433, China; 20112020025@fudan.edu.cn (X.S.); 18112020003@fudan.edu.cn (S.C.); 19112020021@fudan.edu.cn (Y.Z.); 2 School of Microelectronics, Fudan University, Shanghai 200433, China; 3 School of Artificial Intelligence Science and Technology, University of Shanghai for Science and Technology, Shanghai 200093, China; 4 Institute of Photonic Chips, University of Shanghai for Science and Technology, Shanghai 200093, China

**Keywords:** microwave annealing, molybdenum disulfide devices, two dimensional materials, neural network, HSV color

## Abstract

Microwave annealing, an emerging annealing method known for its efficiency and low thermal budget, has established a foundational research base in the annealing of molybdenum disulfide (MoS_2_) devices. Typically, to obtain high-quality MoS_2_ devices, mechanical exfoliation is commonly employed. This method’s challenge lies in achieving uniform film thickness, which limits the use of extensive data for studying the effects of microwave annealing on the MoS_2_ devices. In this experiment, we utilized a neural network approach based on the HSV (hue, saturation, value) color space to assist in distinguishing film thickness for the fabrication of numerous MoS_2_ devices with enhanced uniformity and consistency. This method allowed us to precisely assess the impact of microwave annealing on device performance. We discovered a relationship between the device’s electrical performance and the annealing power. By analyzing the statistical data of these electrical parameters, we identified the optimal annealing power for MoS_2_ devices as 700 W, providing insights and guidance for the microwave annealing process of two-dimensional materials.

## 1. Introduction

Over the past decade, research endeavors have yielded significant advancements in the domain of two-dimensional (2D) materials. These materials, characterized by their layered structure, exhibit remarkable and industrially relevant properties when reduced to a thickness of just a few atomic layers. Molybdenum disulfide (MoS_2_), a quintessential example of 2D materials, has garnered considerable attention for its superior mechanical, optical, and electrical properties [1]. Notably, MoS_2_ is distinguished by its energy bandgap, which ranges between 1.2 and 1.8 eV, offering a distinct advantage over graphene, which lacks an intrinsic bandgap. This characteristic endows MoS_2_ with great potential for application in field-effect transistors (FETs) and other electronic devices.

The Schottky barrier [2] refers to a rectifying barrier formed at the metal–semiconductor interface, characterized by a lower interface voltage and a relatively thin depletion layer at the metal end, affecting current flow and posing an obstacle to device performance. These device performances generally refer to the on/off current, mobility, contact resistance, and subthreshold slope of the device. The Schottky barrier between metal and MoS_2_ presents a significant obstacle to device performance [3], exacerbated by MoS_2_’s propensity to adsorb airborne contaminants due to its two-dimensional nature [4]. This interaction not only compromises device integrity but also hinders its operational efficiency. To mitigate the impact of the metal–MoS_2_ barrier, various strategies have been devised, such as implementing highly doped semiconductor contacts [5] and employing scandium electrodes [6]. However, these solutions face their own sets of challenges: the instability of highly doped contacts and the complexity and cost associated with scandium electrodes limit their industrial applicability. In contrast, annealing techniques have emerged as a widely adopted solution for removing adsorbed impurities from thin film [7]; during annealing, metal atoms are capable of diffusing to the surface or interface region of the semiconductor, filling interface defects. This mechanism enhances the adhesion between the metal and semiconductor, minimizes the density of interface states, and subsequently lowers the contact resistance [8]. Thermal annealing can also reduce these interface defects and states, enhancing carrier transport at the interface [9]. Furthermore, it triggers phase transformations or chemical reactions in the interface region, forming new phases or compounds with lower contact resistance [10]. Techniques such as rapid thermal annealing [11], high-vacuum annealing [12], and laser annealing [8] have been extensively utilized for this purpose, offering a more practical approach to enhancing device performance and longevity.

However, the annealing methods commonly employed are not compatible with flexible MoS_2_ devices, as the typical annealing temperatures exceed 150 °C [13,14]. Such high temperatures can easily damage flexible devices and require prolonged processing times, often exceeding 30 min. Although laser annealing offers the advantage of selective heating, its efficiency is notably low. Moreover, the equipment necessary for laser annealing is costly, rendering it impractical for uniform annealing of devices in large batches. Microwave annealing (MWA), a novel technique emerging in recent years, offers the advantage of a low thermal budget. This method facilitates a reduction in contact resistance for devices and aids in the removal of contaminants adsorbed onto the MoS_2_ channels [15,16,17]. These outcomes stem from the heat generated by electromagnetic loss between the material and the microwaves, similar to the effect of thermal annealing. However, current research on microwave annealing primarily focuses on dopant activation and the formation of ultra-shallow junctions [18], with limited investigations into its effects on MoS_2_ devices.

In the current field of MWA research, the majority of experiments are conducted using limited samples, as demonstrated in studies [15,19]. These investigations have confirmed that microwave annealing has a positive effect on the performance of MoS_2_ devices. However, there is a notable absence of comprehensive research based on large datasets examining the uniform effects of MWA on the performance of MoS_2_ devices. This research gap primarily arises from the challenges associated with fabricating numerous devices and standardizing parameters, which present significant operational difficulties. Particularly in the production of MoS_2_ devices, the inconsistency in film thickness due to the use of the mechanical exfoliation method poses a major issue.

The impact of MWA can vary drastically with differences in film thickness. Thus, ensuring uniform thickness is critical for the reliability of the research findings. CVD can grow uniform MoS_2_ films on large-area substrates, which is essential for mass production and industrial applications [20]. Additionally, CVD process parameters (such as temperature, pressure, gas flow, etc.) can be precisely controlled to adjust the thickness, grain size, and morphology of the MoS_2_ films [21]. Despite CVD’s excellent scalability for industrial-scale production [22], the MoS_2_ films produced by this method may contain more defects and grain boundaries, affecting their electronic and optical properties [23]. In contrast, MoS_2_ sheets prepared by mechanical exfoliation typically exhibit higher crystal quality and lower defect density, making them suitable for research on high-performance electronic devices [24]. Currently, MoS_2_ devices prepared using the mechanical exfoliation method require film thickness to be measured individually using Atomic Force Microscopy (AFM), a process that becomes particularly cumbersome when handling large batches of samples. As a result, visual inspection of film thickness during the exfoliation process is the prevalent method, though this approach relies heavily on human experience and can lead to significant variances in accuracy between individuals. Therefore, there is an urgent need to develop a new technological method to assist in the mechanical exfoliation process, enabling accurate identification and classification of film thickness, thereby improving the accuracy of recognition while reducing experimental costs. Furthermore, clear guidance on how to effectively apply MWA to enhance the performance of MoS_2_-based devices is currently lacking.

In this study, we prepared MoS_2_ devices on a large scale via mechanical exfoliation. It was crucial to collect comprehensive data and maintain consistency in parameters to effectively analyze the impact of microwave annealing. To enhance the accuracy of film thickness identification and reduce the time required for device fabrication, we employed a neural network method based on the HSV color space. This approach not only improved accuracy compared to traditional visual inspection but also significantly reduced the time needed for mass fabrication, ensuring uniformity of the films throughout the experimental process. Subsequently, we subjected the devices to MWA and extracted their electrical performance parameters. Statistical analysis of these parameters allowed us to determine an optimal MWA power setting that significantly enhanced device performance, achieving maximal improvement in their electrical characteristics.

## 2. Experiment

### 2.1. Classification of MoS_2_ Film Thickness

In our experiments, investigating the MWA power for research devices often necessitates the preparation of a large number of devices while ensuring uniformity in channel dimensions and film thickness. Our devices are fabricated using a mechanical exfoliation method from commercial natural MoS_2_ crystals (XFNANO Inc., Nanjing, China, where the thickness of MoS_2_ film under an optical microscope is typically determined by visual inspection. In the process of preparing numerous devices, this step is both tedious and prone to errors. Consequently, there is a need to identify a new method to replace the visual recognition pattern, with the aim of reducing the experimental duration and increasing the accuracy of thickness determination. Human recognition of film thickness primarily relies on the HSV color space model, whereas optical microscopes generally save images in the RGB (red, green, blue) color space model [25]. During the device fabrication process, the identification of MoS_2_ thin films typically involves comparing the optical contrast between the MoS_2_ and the dielectric layer to determine the film’s thickness.

**Figure 1:**
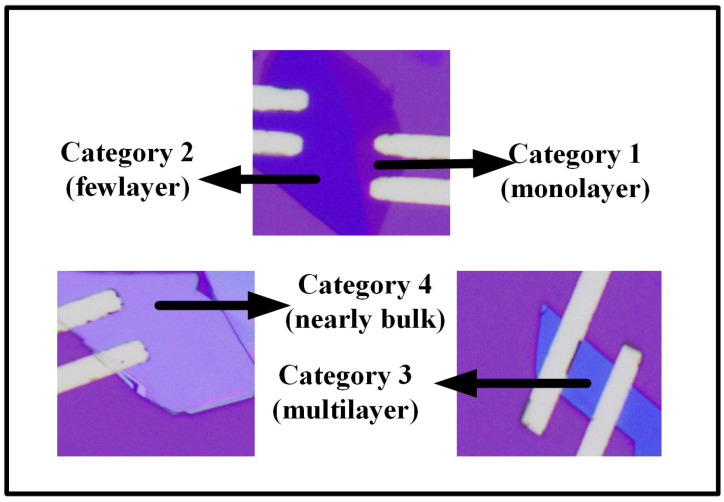
The classification of four typical MoS_2_ thicknesses.

In this experiment, we classified the MoS_2_ films as illustrated in Figure 1 and Table 1. Category 1 consists of monolayer MoS_2_ with a typical thickness measured at approximately 0.65 nm. Category 2 comprises few-layer MoS_2_, where the thickness generally ranges from approximately 1.3 nm to 6.5 nm. Category 3 includes multilayer MoS_2_, with thickness measurements typically falling between approximately 6.5 nm and 32.5 nm. Category 4 features nearly bulk MoS_2_, with a thickness exceeding approximately 32.5 nm. In this study, we chose to fabricate devices using Category 3, specifically, multilayer MoS_2_ films. These films not only maintain the direct bandgap feature intrinsic to MoS_2_ but are also more easily produced via mechanical exfoliation. Additionally, multilayer MoS_2_ films display remarkable chemical stability and reduced sensitivity to environmental changes, making them exceptionally suitable for long-term applications.

### 2.2. Advantages of Using the HSV for Film Thickness Classification

**Figure 2:**
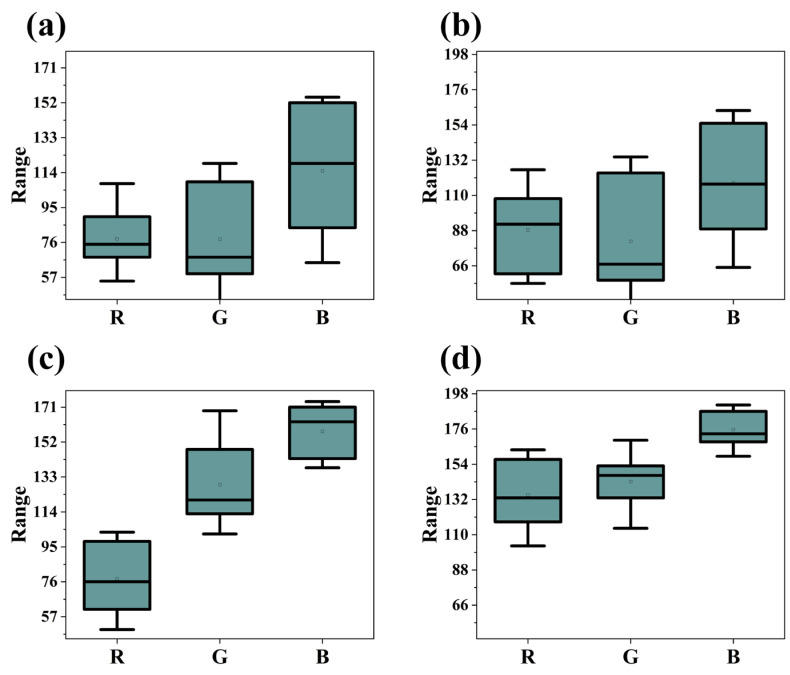
Box-and-whisker plots representing the distribution of RGB values across four distinct categories of film thickness: (**a**) Category 1; (**b**) Category 2; (**c**) Category 3; (**d**) Category 4.

**Figure 3:**
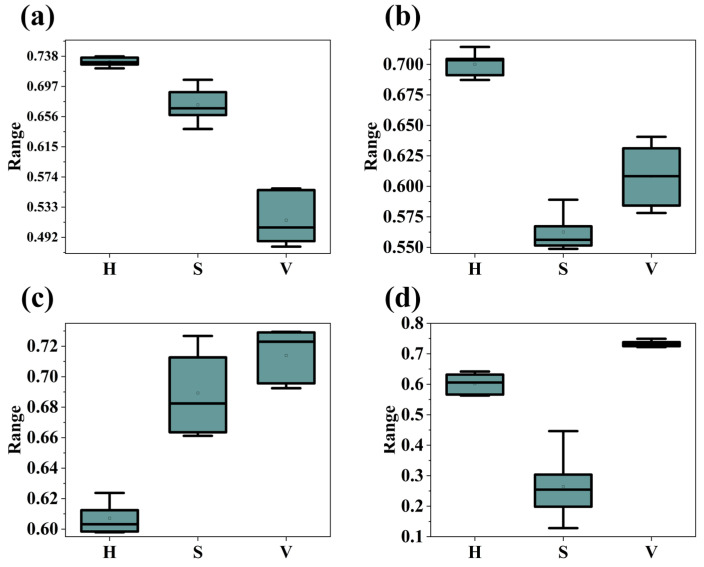
Box-and-whisker plots representing the distribution of HSV values across four distinct categories of film thickness: (**a**) Category 1; (**b**) Category 2; (**c**) Category 3; (**d**) Category 4.

The RGB color space is characterized by an amalgamation of luminance and chromatic information, implying that variations in color within the RGB spectrum concurrently reflect changes in both brightness and hue. A substantial correlation exists among the three channels of RGB, particularly evident within image data. Figure 2 presents box-and-whisker plots for four different categories of MoS_2_ film thicknesses. An examination of Figure 2 reveals that the median values of the red (R) component across all plots are closely aligned, with a similar breadth for the interquartile range, rendering the R value alone insufficient for distinguishing film thicknesses. Additionally, the boxes for the other two components, green (G) and blue (B), exhibit a certain degree of overlap across different film thicknesses. In contrast, the HSV color space aligns more closely with human perception of color by segregating hue (H), saturation (S), and value (V) into distinct components. This separation potentially facilitates a clearer expression of certain color characteristics within the HSV space, as it allows for the independent consideration of hue variations unaffected by changes in luminance. Such independence of parameters might more readily disclose color variations attributed to differences in film thickness. Figure 3 showcases box-and-whisker plots for HSV values across four categories of film thickness. The distributions of the three values are notably concentrated, particularly the hue (H), which exhibits an exceedingly narrow range of hue distribution, with lower degrees of box overlap between different film thicknesses, accentuating the distinctiveness between parameters. Conversely, while the RGB box plots display color variances, these differences may result from a complex interplay of chroma and brightness, complicating the interpretation of film thickness impact directly from the RGB plots. To simulate human recognition patterns, it is essential to convert optical images into the HSV color space [26].

### 2.3. Neural Network Based on HSV Color Space

**Figure 4:**
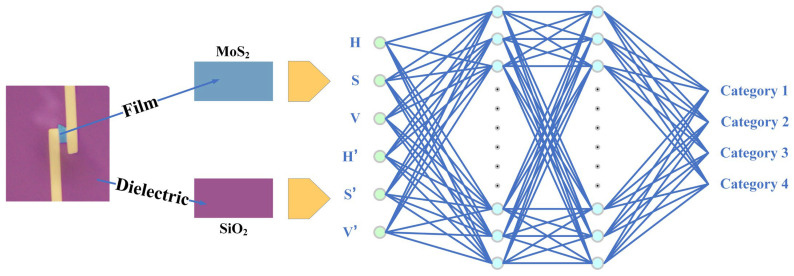
Neural network structure for discriminating membrane thickness.

The second step involves simulating the method by which the human eye discerns film thickness, specifically by comparing the contrast of the film on the substrate to determine the thickness. In this process, we opt to employ a neural network to implement this discrimination method. The structure of the neural network is depicted in Figure 4.

As illustrated in Figure 4, the neural network utilized in this study takes six inputs, which comprise three values from the HSV color space of MoS_2_ thin films and three values from the HSV color space of the silicon dioxide (SiO_2_) dielectric layer adjacent to the films. The neural network features two hidden layers: the first hidden layer contains 128 nodes and employs the Rectified Linear Unit (ReLU) [27] activation function to ensure nonlinear learning capability. The second hidden layer has its number of nodes halved to 64, also utilizing the ReLU activation function. Given the Adam optimizer’s effective performance on such problems [28,29], it was selected as the optimizer for this network, with a learning rate set at 0.001. The dataset was divided, allocating 80% for training and the remaining 20% for validation; the neural network in this article is based on PyTorch of python (The version of Python is 3.11.5).

### 2.4. Classification Results

**Figure 5:**
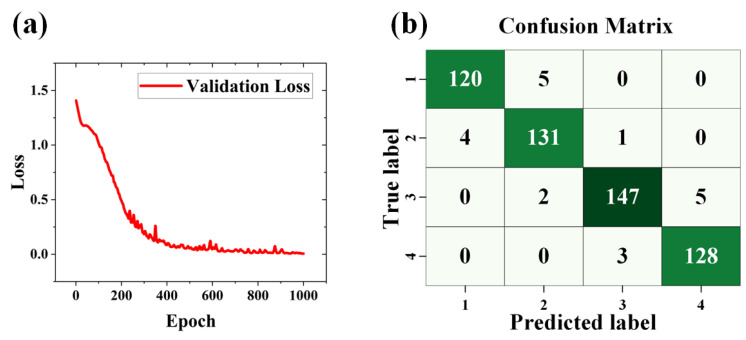
Showcased the training process and results of the neural network: (**a**) A graph depicting the change in validation loss over epochs. (**b**) The confusion matrix of prediction results; 1, 2, 3, and 4 indicate Category 1, Category 2, Category 3, and Category 4.

Figure 5a illustrates the curve depicting how the neural network’s loss on the validation set changes with epochs. From the figure, it is evident that the loss decreases progressively with an increasing number of training epochs, indicating a positive training trend. Figure 5b presents the confusion matrix of the prediction results. Within the confusion matrix, most predictions are concentrated along the diagonal, signifying that the model accurately classifies the majority of the samples. High values on the diagonal and low values of the diagonal demonstrate the model’s strong performance across all categories, indicating excellent predictive outcomes of the network. This approach significantly reduces the time required for device fabrication compared to manual thickness identification by the human eye while also achieving high accuracy.

### 2.5. Device Fabrication and MWA

The fabrication of multilayer MoS_2_ transistors begins with the preparation of designated substrates. This involves utilizing heavily doped p-type silicon wafers, which are covered with thermally oxidized SiO_2_ dielectric layers (the thickness is 230 nm). These wafers are then segmented into 1 cm × 1 cm squares, each marked for identification. Few-layer MoS_2_ samples are procured via mechanical exfoliation and are subsequently transferred onto these marked squares. Prior to this transfer, the squares are meticulously cleaned using acetone, alcohol, and deionized water to ensure a contaminant-free surface. Under microscopic observation, the positions of the multilayer MoS_2_ samples on the squares are accurately determined. The construction of multilayer MoS_2_ transistors on these substrates is achieved through a sequence of processes, including electron beam lithography (the electron beam lithography machine used in this experiment is the JBX-6300FS, JEOL, Tokyo, Japan), physical vapor evaporation (the physical vapor deposition machine used in this experiment is the PVD 75, Kurt J. Lesker, Shanghai, China, and lift-off techniques. Chromium/gold (Cr/Au) electrodes, with thicknesses of 20/96 nm, respectively, are employed as the source–drain electrodes. To enhance device performance, MWA is applied using the AXOM-300 MWA system from DSG Company (San Jose, CA, USA). This system operates at a microwave magnetron frequency of 5.8 GHz.

Subsequently, MWA was conducted in a nitrogen atmosphere using varying microwave power levels (210 W, 490 W, 700 W, and 980 W) over a period of 600 s. Electrical evaluations were performed before and after MWA using an Agilent B1500A semiconductor characterization system (Agilent Technologies, Shanghai, China) situated within a shielded probe station at room temperature. Following the annealing process, post-annealing assessments were carried out once the device had returned to ambient temperature.

In our experiment, to ensure the consistency and reliability of the experimental data, we endeavored to maintain uniformity in both the thickness of mechanically exfoliated MoS_2_ films and the dimensions of the channels across all devices. The method to achieve uniform film thickness involves utilizing neural networks to determine the thickness, a process that has been detailed extensively in prior discussions. Attaining uniformity in channel dimensions is relatively straightforward, which is accomplished by replicating the same pattern during electron beam lithography. The statistical data pertaining to the film thickness and channel dimensions of MoS_2_ devices are presented in Table 2.

## 3. Results and Discussion

Table 3 presents the temperature variations observed during various MWA procedures, as measured by an infrared thermometer positioned underneath the equipment. Within a nitrogen atmosphere, the recorded peak temperatures corresponding to microwave powers of 210 W, 490 W, 700 W, and 980 W are 60.1 °C, 87.5 °C, 121.2 °C, and 148.8 °C, respectively. From Table 3, it is evident that the peak temperatures achieved during our MWA process are consistently lower than the glass transition temperatures commonly associated with flexible semiconductor substrates, such as polyimide (PI) and polyethylene naphtholate (PEN).

**Figure 6:**
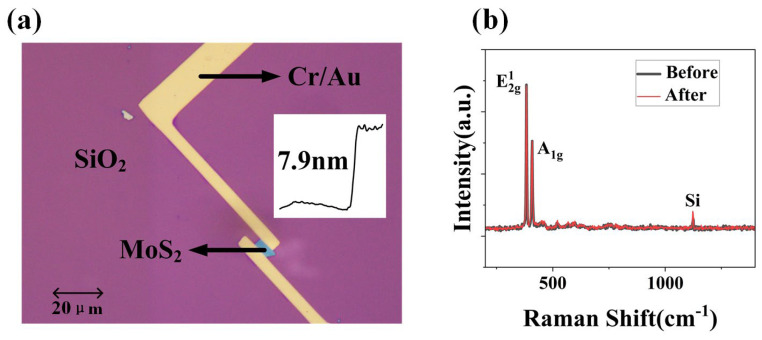
(**a**) Optical photograph of a typical MoS_2_ device, accompanied by precise values measured via AFM before (in black) and after (in red). The scale bar in the figure is 20 μm. (**b**) Raman spectroscopy profiles before (in black) and after (in red) 700 W MWA power.

To evaluate the impact of MWA at varying power levels on the performance of MoS_2_ transistors, we performed experiments to examine the transfer characteristics of multilayer MoS_2_ transistors as influenced by the annealing power. As illustrated in Figure 6, Figure 6a presents an optical photograph of a typical MoS_2_ device, accompanied by precise thickness measurements obtained through AFM; the exact value of its film thickness is 7.9 nm. Figure 6b displays Raman spectroscopy characterizations of the device before and after annealing, with the black line representing pre-annealing and the red line post-annealing. From the Raman spectroscopy characterization, we can observe that the peak positions for $E_{2g}^{1}$ and $A_{1g}$ are at 382.6 cm^−1^ and 408.4 cm^−1^, respectively, with a difference of 25.8 cm^−1^. This difference characterizes the features of multilayer MoS_2_ film. Additionally, a lower peak position at 1122.5 cm^−1^ signifies the peak corresponding to silicon (Si). From the Raman spectroscopy analysis conducted before and after annealing, it is observable that there are virtually no changes in the peak values. This comparison suggests that MWA in a nitrogen atmosphere has a minimal impact on the structural integrity of the multilayer MoS_2_ film, as demonstrated by the negligible differences in the Raman spectra pre- and post-annealing [30].

### 3.1. Results of Transfer and Output Characteristic Curves

**Figure 7:**
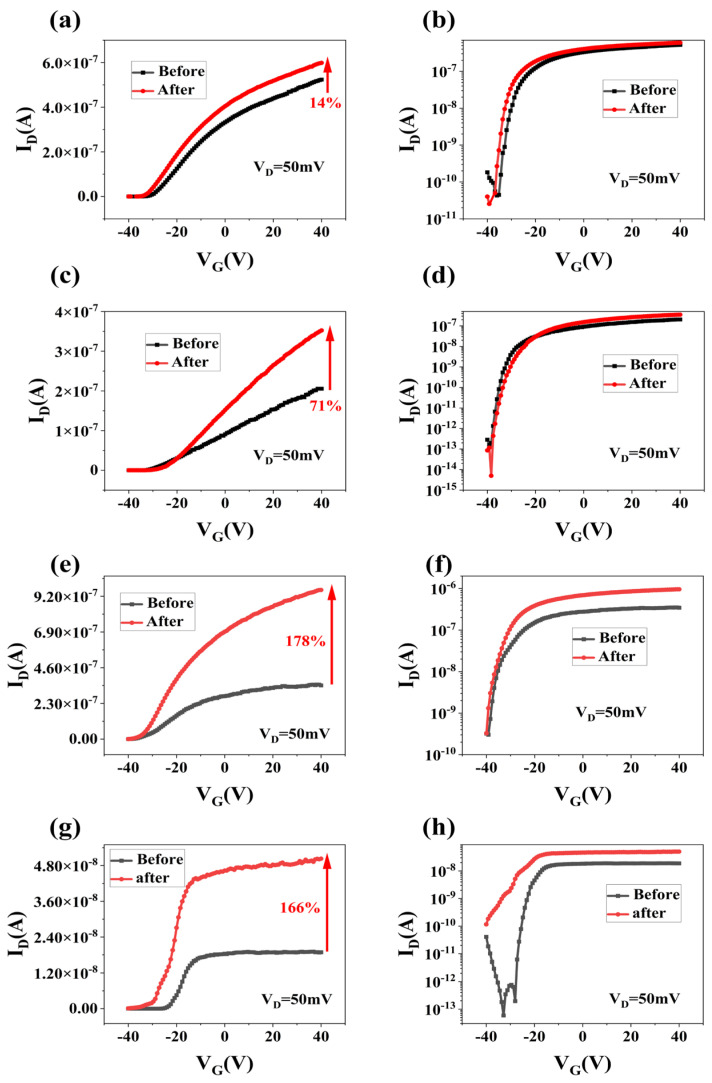
(**a**,**b**), respectively, depict the transfer characteristic curve and the logarithmic form of the representative MoS_2_ FET following exposure to 210 W MWA power in an N_2_ atmosphere. (**c**,**d**), respectively, depict the transfer characteristic curve and the logarithmic form of the representative MoS_2_ FET following exposure to 490 W MWA power in an N_2_ atmosphere. (**e**,**f**), respectively, depict the transfer characteristic curve and the logarithmic form of the representative MoS_2_ FET following exposure to 700 W MWA power in an N_2_ atmosphere. (**g**,**h**), respectively, depict the transfer characteristic curve and the logarithmic form of the representative MoS_2_ FET following exposure to 980 W MWA power in an N_2_ atmosphere.

**Figure 8:**
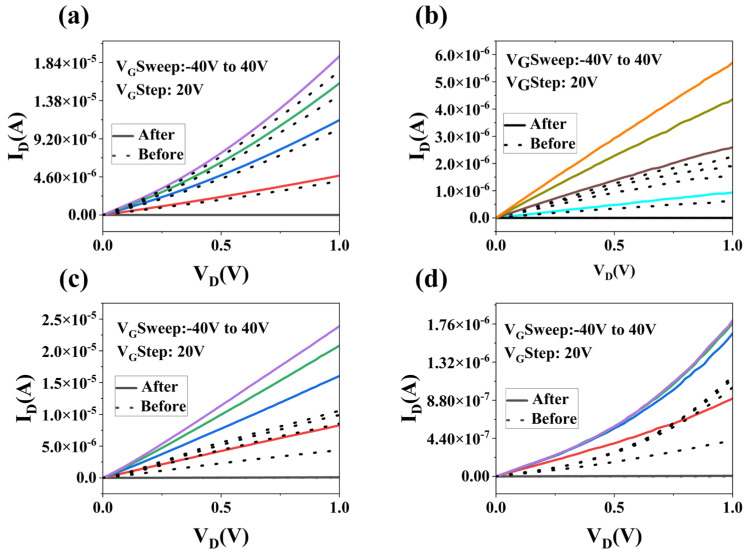
The curve before annealing is represented by a dotted line, and the curve after annealing is represented by a solid line. (**a**) The output curve of the representative MoS_2_ FET after the 210 W MWA powers in the N_2_ atmosphere. (**b**) The output curve of the representative MoS_2_ FET after the 490 W MWA powers in the N_2_ atmosphere. (**c**) The output curve of the representative MoS_2_ FET after the 700 W MWA powers in the N_2_ atmosphere. (**d**) The output curve of the representative MoS_2_ FET after the 980 W MWA powers in the N_2_ atmosphere.

Figure 7 and Figure 8 present the transfer characteristic curves and output characteristic curves of typical multilayer MoS_2_ transistors under four different annealing power settings. The transfer characteristic curves clearly demonstrate n-type behavior in the channels of the fabricated multilayer MoS_2_ transistors, indicating that the drain current increases with the rise in gate voltage. Figure 7 presents schematic diagrams of the typical transfer characteristics of MoS_2_ at varying power levels in our dataset. From the transfer characteristic curves shown in Figure 7a,b, we observe an increase in the on-state current from $5.23\times 10^{-7}\;A$ to $5.98\times 10^{-7}\;A$, a 14% improvement, under 210 W annealing power. Similarly, at a power level of 490 W, shown in Figure 7c,d, the on-state current increased from $2.06\times 10^{-7}\;A$ to $3.52\times 10^{-7}\;A$, marking a 71% improvement. The enhancement is even more pronounced at 700 W power, where the on-state current escalated from $3.46\times 10^{-7}\;A$ to $9.61\times 10^{-7}\;A$, an increase of 178% in Figure 7e,f. Although at 980 W power, the on-state current rose from $1.89\times 10^{-8}\;A$ to $5.03\times 10^{-8}\;A$, a 166% increase, the improvement is still substantial in Figure 7g,h.

However, it is important to note that the off-state current also experienced a significant rise at this level. The off-state current increased from $1.95\times 10^{-13}$ A to $1.16\times 10^{-10}$ A, leading to a severe degradation in the on/off ratio; the on/off ratio is only $4.34\times 10^{2}$ after microwave annealing. From the transfer characteristic curves of MoS_2_ devices, it is evident that MWA has a significant positive correlation with the device’s on-state current. This means that as the annealing power increases, the on-state current also gradually increases. However, an increase in annealing power is not always beneficial. Specifically, when the annealing power exceeds 700 W, there is a noticeable increase in the off-state current, which is disadvantageous for achieving an ideal on/off ratio in the device.

Figure 8 illustrates the output characteristic curves of MoS_2_ devices before and after MWA at various power levels. It is evident from the figure that, under the same gate voltage, the source–drain current increases post-annealing compared to its pre-annealed state, with the most significant enhancements observed following annealing at 700 W and 980 W microwave power. This observation is consistent with the analysis of the transfer characteristic curves presented in Figure 8, underscoring the pronounced effect of MWA on enhancing the on-state current.

### 3.2. Results of Other Electrical Parameters

**Figure 9:**
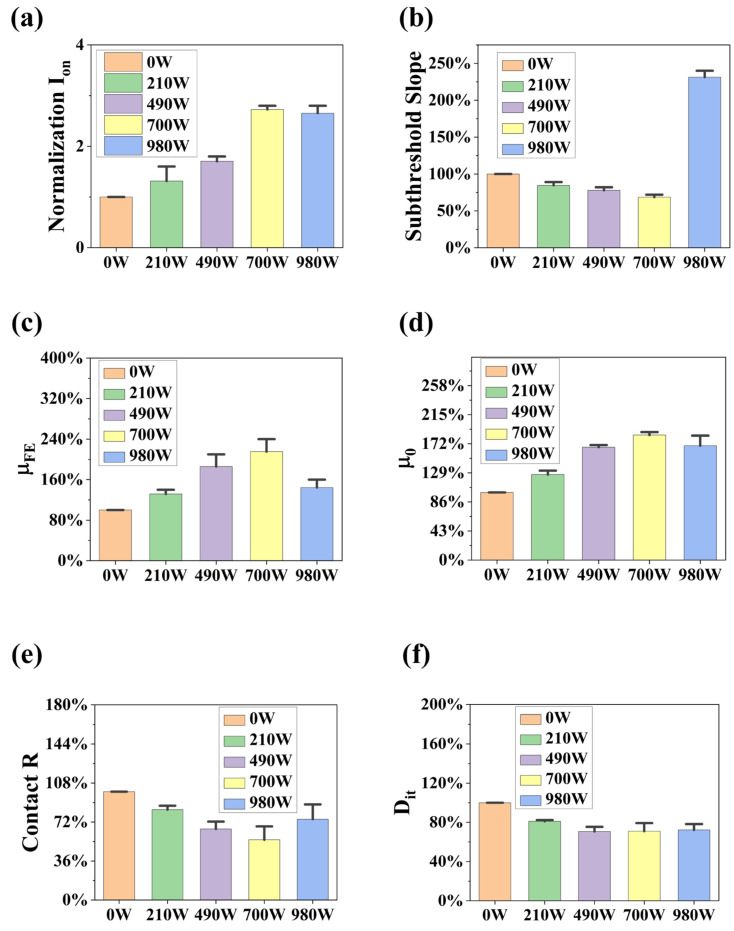
(**a**) Statistical data on the on-state current. (**b**) Statistical data on the subthreshold slope. (**c**) Statistical data on the field-effect mobility. (**d**) Statistical data on the intrinsic mobility. (**e**) Statistical data on the contact resistance. (**f**) Statistical data on the trap density.

Additionally, we evaluated the average of the normalized on-state current across four few-layer MoS_2_ transistors subjected to annealing in an N_2_ atmosphere as a function of the annealing power, depicted in Figure 9a. The on-state current is identified as the maximal output current achieved within a high gate voltage range. The reference point for normalizing the on-state current is its value for the corresponding multilayer MoS_2_ transistor prior to annealing. It is evident that the on-state current of MoS_2_ devices increases with escalating annealing power, indicating a positive correlation between MWA and the on-state current of the devices. Subsequently, we investigated the relationship between the subthreshold swing (SS), defined as $SS=\frac{dV_{g}}{d({log}_{10}I_{d})}$, and the annealing power. Figure 9b showcases statistical data across various microwave powers, and it reveals that the subthreshold swing decreases with increasing annealing power; however, a notable increase in the subthreshold swing is observed when the annealing power exceeds 700 W, indicating a deterioration in device performance at excessively high annealing powers.

Further investigations were conducted on the variation in field-effect and intrinsic mobility with annealing power; field-effect mobility is a critical parameter for assessing the performance of MOSFETs. A high field-effect mobility indicates that the MOSFET possesses superior switching speeds and lower power consumption. The field-effect mobility can be calculated using the following equation [16,31]:

(1) $$\mu_{eff}=\frac{Lg_{m}}{WC_{ox}V_{DS}}$$

Compared to field-effect mobility, intrinsic mobility values are not influenced by contact resistance. Consequently, intrinsic mobility can be utilized to determine whether the MWA effects on MoS_2_ transistors occur at the metal–semiconductor interface or within the MoS_2_ channel itself. The value of intrinsic mobility can be calculated using the Y-function formula described in the literature [8]:

(2) $$Y=\frac{I_{ds}}{\sqrt{g_{m}}}=\sqrt{\frac{W}{L}\mu_{0}C_{ox}V_{ds}}\cdot \left(V_{gs}-V_{th}\right)$$

Figure 9c,d display the statistical distribution of field-effect mobility and intrinsic mobility data. It can be observed that the field-effect mobility gradually increases with the rise in annealing power until it reaches 700 W, beyond which it begins to decline. The trend in intrinsic mobility mirrors that of field-effect mobility, albeit with a relatively lower rate of increase. This indicates that the impact of MWA on the metal–semiconductor contact area is significantly greater than its effect on the MoS_2_ channel. There are two main reasons for this. Firstly, MoS_2_ is a material with low electromagnetic loss. Thus, it does not generate heat through electromagnetic losses in a microwave field; its temperature primarily relies on thermal conduction. Secondly, the MoS_2_ thin films in our experiment were prepared using a mechanical exfoliation technique, ensuring high film quality from the start. The annealing process primarily serves to remove contaminants adsorbed due to the two-dimensional nature of the material.

To further analyze the impact of MWA on device performance, we extracted trends in contact resistance and trap density as functions of annealing power. Figure 9e,f display the statistical data of contact resistance and trap density variations with annealing power, respectively. The generation of contact resistance primarily arises from the interaction between metals and n-type semiconductors. This is due to the work function of metals often being lower than that of semiconductors, leading to a flow of electrons from the semiconductor to the metal to balance the Fermi levels. This process results in the formation of a depletion zone at the semiconductor surface where electron concentration is diminished, subsequently creating an energy barrier known as the Schottky barrier. In the off and on states of MoS_2_ transistors, thermionic emission and tunneling through the Schottky barrier, respectively, restrict charge injection. As observed in Figure 9e, the contact resistance initially shows a decreasing trend with increasing microwave power until it surpasses 700 W, after which it begins to increase. This pattern aligns with our previous analysis of mobility changes, further validating the accuracy of the annealing trend. According to the output characteristics curve and statistical data on contact resistance, the reduction in contact resistance is primarily attributed to MWA potentially increasing the carrier concentration at the interface, thereby reducing the width of the tunneling barrier. The explanation for this phenomenon is discussed in [8], mainly attributed to the post-annealing diffusion of Cr into MoS_2_, forming a Cr-MoS_2_ solid solution. The doped MoS_2_ becomes more conductive as a result. The reduction in the tunnel barrier width by forming such solid solutions at the interface is the primary mechanism behind the decrease in contact resistance following microwave annealing. When microwave power exceeds 700 W, an increase in contact resistance is observed, potentially due to the high microwave power causing temperature increases that damage the metal–semiconductor interface. Although the measured temperatures are not high, the unique loss mechanism of microwaves in a 5.8 GHz electromagnetic environment leads to the metal electrodes, which are thinner than the skin depth, becoming efficient microwave absorbers [16]. As the microwave power gradually increases, the electrode temperature may exceed the tolerance temperature of MoS_2_, thereby damaging the metal–semiconductor interface. The formula for calculating trap density is derived from the literature [17], and the results shown in Figure 9f, we observed that variations in trap density across different microwave power settings were not significant; a modest decrease in trap density was noted as the annealing power increased. Since the variation in trap density was not significantly evident, and considering the substantial correlation between trap density and the interface of the dielectric layer with the MoS_2_ film, we further characterized the dielectric layer before and after annealing (700 W) to investigate the differences in the surface of SiO_2_ dielectric layer pre- and post-annealing.

### 3.3. AFM Results of the SiO_2_ Dielectric

**Figure 10:**
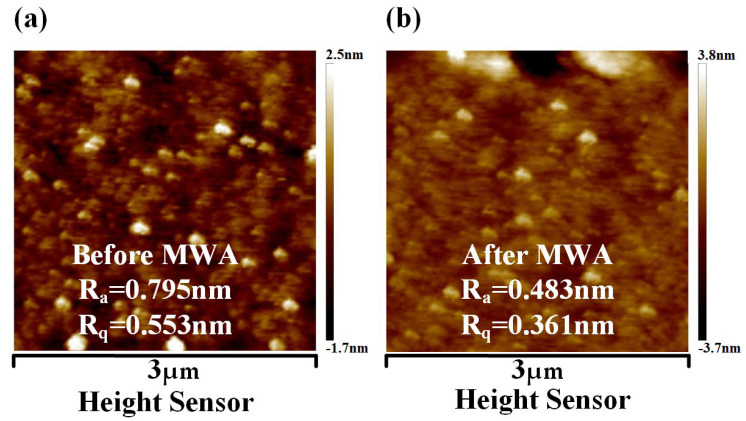
(**a**) AFM characterization of SiO_2_ dielectric without MWA. (**b**) AFM characterization of SiO_2_ dielectric after MWA at 700 W.

Figure 10 presents the AFM images of a typical SiO_2_ dielectric layer before and after annealing. From the AFM images, it can be observed that the roughness average (Ra) value decreased from 0.795 nm before annealing to 0.483 nm afterward, and the root mean square (Rq) value dropped from 0.553 nm to 0.361 nm post-annealing. This suggests a minor improvement in the surface quality of the SiO_2_ dielectric layer due to annealing, although the changes are almost negligible. This phenomenon is understandable, given that silicon dioxide is not an effective microwave-absorbing material. Therefore, it does not generate heat from electromagnetic loss in a microwave field. Only the thermal conduction at the metal–semiconductor junction has a certain impact on the nearby SiO_2_ substrate layer. We hypothesize that the significant enhancement observed in the SiO_2_ dielectric layer following MWA is primarily attributable to the elimination of impurities. These impurities, predominantly residues such as adhesives from mechanical exfoliation, are effectively removed during the process. Additionally, the procedure also eliminates water molecules adsorbed on the surface of MoS_2_ [30]. Consequently, the impact of MWA on trap density is also extremely limited.

## 4. Conclusions

In this experiment, we introduced a neural network approach based on the HSV color space inspired by human methods of recognizing the thickness of MoS_2_ films. This technique significantly enhances the accuracy of thickness identification during the mechanical exfoliation of large batches of devices, improving experimental efficiency and reducing the difficulty of generating extensive datasets. This method supports a comprehensive statistical analysis of the impact of MWA on the performance of MoS_2_ devices. We conducted a comparative analysis of data before and after annealing for devices subjected to microwave powers of 210 W, 490 W, 700 W, and 980 W, focusing on the statistical data related to their electrical parameters. Our findings demonstrate an improvement in device performance that correlates with an increase in annealing power, with the peak enhancement in the on-state current occurring at 700 W. This result aligns with findings from other researchers. Other electrical performance metrics also achieved optimal levels at this power setting. Through this study, we have identified the optimal annealing power for molybdenum disulfide devices via MWA and provided guidelines for the annealing process. Additionally, our method for thickness recognition does not rely on the material properties but solely on the optical color space, suggesting potential applicability for thickness recognition in other two-dimensional materials, thereby aiding in the preparation of large datasets for other two-dimensional devices.

## Figures and Tables

**Table 1:** Statistical parameters of MoS_2_ films.

	Category 1	Category 2	Category 3	Category 4
Thickness (nm)	≈0.65	≈1.3 to 6.5	≈6.5 to 32.5	More than 32.5

**Table 2:** Statistical parameters of MoS_2_ devices.

The Mean of Film Thickness (nm)	The Variance of Film Thickness (nm^2^)	Channel Length (μm)	Channel Width (μm)
7.9	0.11	3.3 ± 0.2	3.8 ± 0.2

**Table 3:** Peak temperature corresponding to different MWA power.

MWA Power	210 W	490 W	700 W	980 W
Temperature	60.1 °C	87.5 °C	121.2 °C	148.8 °C

## Data Availability

The original contributions presented in the study are included in the article, further inquiries can be directed to the corresponding authors.
